# Na^+^ dependent acid-base transporters in the choroid plexus; insights from *slc4* and *slc9* gene deletion studies

**DOI:** 10.3389/fphys.2013.00304

**Published:** 2013-10-22

**Authors:** Henriette L. Christensen, An T. Nguyen, Fredrik D. Pedersen, Helle H. Damkier

**Affiliations:** Department of Biomedicine, Aarhus University - HealthAarhus, Denmark

**Keywords:** cerebrospinal fluid, brain pH, knockout mice, membrane transporters, epithelial physiology

## Abstract

The choroid plexus epithelium (CPE) is located in the ventricular system of the brain, where it secretes the majority of the cerebrospinal fluid (CSF) that fills the ventricular system and surrounds the central nervous system. The CPE is a highly vascularized single layer of cuboidal cells with an unsurpassed transepithelial water and solute transport rate. Several members of the *slc4a* family of bicarbonate transporters are expressed in the CPE. In the basolateral membrane the electroneutral Na^+^ dependent Cl^−^/HCO_3_^−^ exchanger, NCBE (*slc4a10*) is expressed. In the luminal membrane, the electrogenic Na^+^:HCO_3_^−^ cotransporter, NBCe2 *(slc4a5)* is expressed. The electroneutral Na^+^:HCO_3_^−^ cotransporter, NBCn1 (*slc4a7*), has been located in both membranes. In addition to the bicarbonate transporters, the Na^+^/H^+^ exchanger, NHE1 (*slc9a1*), is located in the luminal membrane of the CPE. Genetically modified mice targeting *slc4a2*, *slc4a5*, *slc4a7*, *slc4a10*, and *slc9a1* have been generated. Deletion of *slc4a5, 7* or *10*, or *slc9a1* has numerous impacts on CP function and structure in these mice. Removal of the transporters affects brain ventricle size (*slc4a5* and *slc4a10*) and intracellular pH regulation (*slc4a7* and *slc4a10*). In some instances, removal of the proteins from the CPE (*slc4a5, 7*, and *10*) causes changes in abundance and localization of non-target transporters known to be involved in pH regulation and CSF secretion. The focus of this review is to combine the insights gathered from these knockout mice to highlight the impact of *slc4* gene deletion on the CSF production and intracellular pH regulation resulting from the deletion of *slc4a5, 7* and *10*, and *slc9a1*. Furthermore, the review contains a comparison of the described human mutations of these genes to the findings in the knockout studies. Finally, the future perspective of utilizing these proteins as potential targets for the treatment of CSF disorders will be discussed.

## Introduction

Membrane transporters of the *slc4* and *slc9* family are expressed in many tissues mediating numerous functions including acid-base regulation and movement of large amounts of fluid and solutes.

The Na^+^ dependent acid-base transporters, s*lc4a5*, −*7*, −*10*, and −*11*, and *slc9a1* are all expressed in the choroid plexus epithelium (CPE) (Praetorius et al., 2004a; Bouzinova et al., 2005; Damkier et al., 2007; Damkier and Praetorius, 2012) where they, in addition to intra- and extracellular acid-base regulation, are involved in cerebrospinal fluid (CSF) secretion.

Over the last decades many genetically modified mouse models have been developed targeting these transporters. The phenotypes reported in some of these mice highlight the significance of the transporters in the CPE. The objective of the current review is to extract the insights gathered from these studies to give an overview of the important role of Na^+^ dependent pH regulators in the CPE. Removal of a protein by genetic modification in animal models gives an indication of the consequences of genetic mutations in human. It does, however, not necessarily provide insight into the physiological role of the transporter in the tissue. An important lesson from the studies of knockout mice is the compensatory mechanisms that take place when removing a protein by genetic modification. This makes it difficult to extrapolate the role of the isolated protein in the tissue. This role is potentially not the same as that observed when inhibiting the protein pharmacologically. In this review we will attempt to extract the data from the knockout mice and compare these to the human mutations and pharmacological studies of these transporters in the CPE and finally relate to studies of the same transporters in other epithelia.

## Choroid plexus structure and morphology

**Figure 1:**
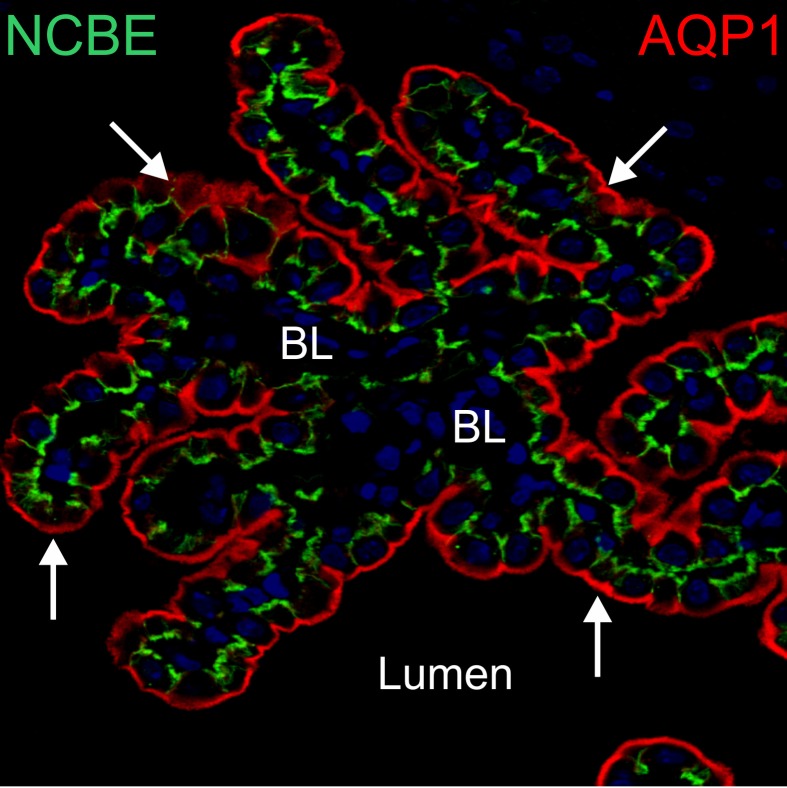
**AQP1 and NCBE in the choroid plexus.** Confocal micrograph of mouse choroid plexus stained with anti-AQP1 antibodies (red) and anti-NCBE antibodies (green). AQP1-specific labeling is seen in the luminal membrane (arrows). NCBE-specific labeling is seen in the basolateral membrane and with high abundance also in the basal labyrinth between adjoining cells. BL: basolateral. Lumen: 4th ventricle lumen. This illustration was published by Damkier *et al*. (2012) (Damkier and Praetorius, 2012).

The CPE constitutes a specialized and important part of the blood-CSF barrier (BCSFB). This barrier consists of endothelium, endothelial basement membrane, interstitial space with sparse connective tissue, subepithelial basement membrane and a single layer of cuboidal epithelial cells (Figure 1).

During fetal development, certain areas of the brain ependyma become highly vascularized (Zagorska-Swiezy et al., 2008) and form the specialized secretory epithelium, protruding into the ventricles. The CPE is found in all four ventricles of the brain, forming branched, villous structures.

In contrast to the vasculature that constitutes the tight blood-brain barrier, the capillaries of the CPE are fenestrated and thus the fluid in the interstitial space resembles plasma ultra-filtrate. In fact, molecules as large as ferritin (~450kDa) are able to pass across the endothelium (Hurley et al., 1981). The permeability of the CP vasculature may be subject to regulation, as vascular endothelial growth factor secreted by CP epithelial cells both maintain (Kamba et al., 2006; Maharaj et al., 2008) and induce (Esser et al., 1998) the formation of fenestrations in endothelium.

The barrier function of the BCSFB is therefore mainly generated through the tightness of the CPE, as the single layer of cells is sealed by tight junctions (Wolburg et al., 2001). This is in contrast to the blood-brain barrier where the barrier function is made up of the tight junctions in the endothelial cells of the vascular wall (Figure 3). The basolateral membrane of the CPE is highly convoluted and intertwining, allowing for rapid transport of solutes to and from the cytoplasm. The apical membrane of the epithelium is covered with microvilli, enlarging the cell surface area and facilitating the role of the epithelium as a highly secretory organ and regulator of CSF composition. The CPE cells also contain clusters of cilia (Mestres et al., 2011), critical for maintaining the flow and possibly the secretion of CSF (Narita et al., 2010).

## Cerebrospinal fluid secretion

The main role of the CPE is to produce CSF. The majority (approximately 80%) of the CSF is secreted by the CPE while the remaining 20% is derived from the brain interstitial fluid (Redzic et al., 2005). The adult human CPE secretes approximately 0.5 L CSF/day at a rate of approximately 0.2–0.4 ml/min/g tissue. The total CSF volume surrounding the brain and spinal cord is estimated to be about 150 ml in adults and is distributed in the cranial and spinal subarachnoid spaces (125 ml) and in the brain ventricles (25 ml) (Sakka et al., 2011). The [Na^+^]_CSF_ in rabbit was measured to be 149 mM compared to 148 mM in plasma. The [HCO_3_^−^]_CSF_ in rabbit was estimated to 22 mM compared to 25 mM in plasma (Davson and Segal, 1996). The composition of the ions in the CSF differs from that predicted for a plasma ultrafiltrate as Na^+^, Mg^++^, and HCO_3_^−^ concentrations are higher and K^+^ and Ca^++^ are lower in CSF (Ames et al., 1964). Thus, the CSF is actively secreted by the CPE and the concentrations of these ions stay relatively constant despite fluctuations in plasma (Husted and Reed, 1977; Murphy et al., 1986). Finally, CSF is slightly hypertonic compared to plasma (approximately 5 mM CSF positive, Davson and Purvis, 1954) which would not be expected from an ultra-filtrate.

**Figure 2:**
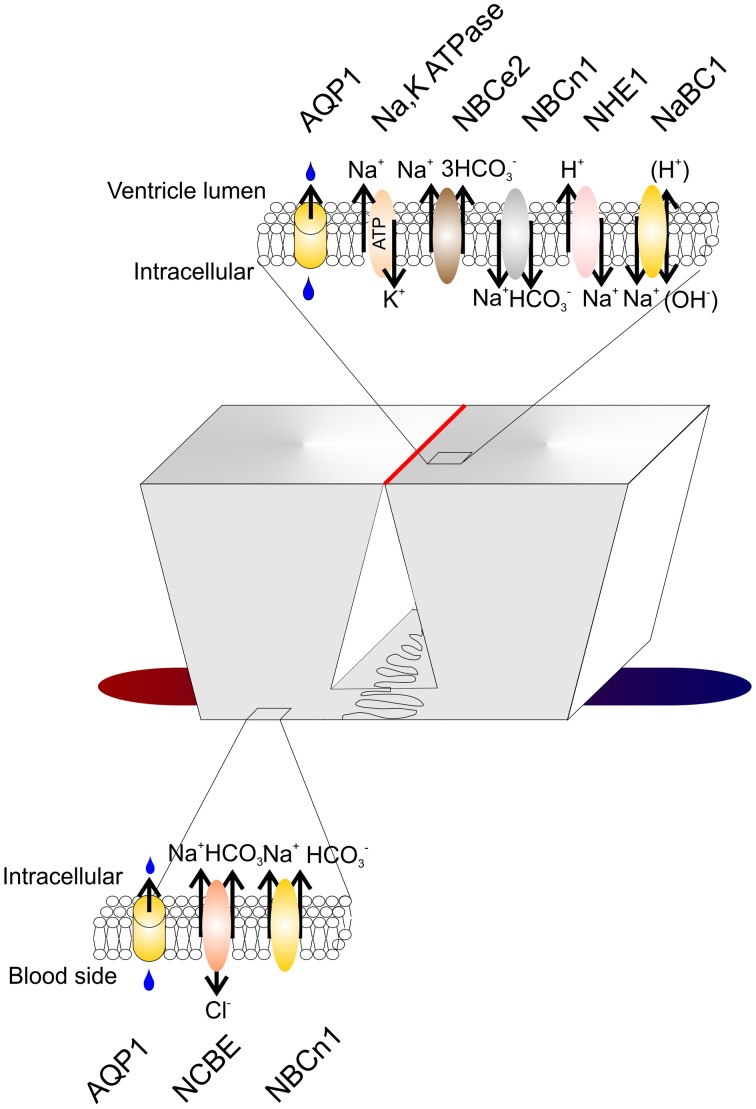
**Transporters in the choroid plexus.** Schematic presentation of two choroid plexus epithelial cells residing on a vascular bed (red to blue). The basolateral membrane is characterized by lateral infoldings near the basement membrane. The wider luminal membrane is connected with tight junctions (red line). Top and bottom: Membrane transporters in the luminal and basolateral membrane (see text for details). AQP1, Aquaporin-1; NBCe2, Electrogenic Na^+^ HCO_3_^−^ co-transporter; NBCn1, Electroneutral Na^+^ HCO_3_^−^ co-transporter; NHE1, Na^+^/H^+^ exchanger; NaBC1, Na^+^:B(OH)^−^_4_ co-transporter; NCBE, Na^+^ dependent Cl^−^/HCO_3_^−^ exchanger.

CSF secretion by the CPE is unlike in other secretory epithelia driven by an osmotic gradient generated by the transcellular movement of Na^+^ from blood through CPE to the CSF (Wright, 1972). In other secretory epithelia, Cl^−^ drives secretion. The osmotic gradient drives water to be moved from plasma to the CSF. Unlike most other secretory epithelia the Na, K ATPase is located in the luminal membrane of the CPE (Figure 2). The Na, K ATPase is pivotal for creating the Na^+^ gradient that drives Na^+^ import across the basolateral membrane (Ames et al., 1965). Na^+^ enters the cell presumably via the Na^+^ dependent HCO_3_^−^ co-transporter, NCBE. The movement of water through the CPE is mainly believed to be mediated by AQP1 located in the luminal membrane and to a smaller degree in the basolateral membrane (Figure 1) (Nielsen et al., 1993; Praetorius, 2007). Some water-movement could also occur through a paracellular route, e.g., via claudin-2 (Rosenthal et al., 2010).

## Regulation of pH in brain and CSF

Acid-base balance in the extracellular fluid surrounding the brain is a prerequisite for optimal brain function, as the neurons are very sensitive to changes in pH (Balestrino and Somjen, 1988). The blood brain barrier (BBB) is virtually impermeable to HCO_3_^−^ and H^+^ (De Bersaques and Leusen, 1954; Siesjo and Ponten, 1966) while CO_2_ readily diffuses across the BBB into the brain parenchyma (Ponten, 1964; Geiser et al., 1996). CO_2_ from the blood is in equilibrium with the CO_2_ in the CSF, and numerous studies have shown that changes in arterial pCO_2_ affect CSF pCO_2_, pH, and ventilation (Pappenheimer et al., 1965; Fencl et al., 1966; Loeschcke and Sugioka, 1969; Andrews et al., 1994). Furthermore, it is known that neuronal excitability is enhanced by increases in brain pH, and decreased when brain pH is lowered (Balestrino and Somjen, 1988). A pioneering study showed that rat pups exposed to hyperthermia developed respiratory alkalosis and febrile seizures (Schuchmann et al., 2006). Seizures have long been known to be halted by inhalation of CO_2_ (Pollock et al., 1949). CSF is practically devoid of proteins, meaning that the non-HCO_3_^−^ buffering power is almost non-existing, and, as the BBB is impermeable to HCO_3_^−^ and H^+^, the brain must rely on other mechanisms for regulating its acid-base balance in the face of changes in plasma pCO_2_. Apart from the ventilatory response upon changes in CSF/extracellular acid-base status, it is believed that the CPE plays a role in the regulation of CSF pH by secretion and absorption of acid/base equivalents. In 1967, Kazemi showed an almost identical increase in arterial blood pCO_2_ and CSF pCO_2_ in dogs after 1 h of 10% CO_2_ inhalation. In the dogs that hyperventilated, similar drops in pCO_2_ were observed in blood and CSF. The ensuing changes in CSF pH were of the same magnitude as the changes in blood pH in both experiments after 1 h. However, 6 h into the experiment, CSF pH had returned toward its resting value, whereas no similar compensation was observed in the blood (Kazemi et al., 1967). This indicates that CSF pH is somehow protected against long-term acid-base disturbances. Furthermore, CO_2_-stimulated increases in CSF [HCO_3_^−^] are accompanied by an increase in CSF [Na^+^] (Nattie, 1980), indicating that buffering of CSF pH is Na^+^-dependent. As the CPE expresses a range of Na^+^ dependent acid-base transporters (Figure 2) it is therefore well equipped for protecting CSF pH against the effects of changes in blood pCO_2_.

## Basolateral Na^+^ dependent acid-base transporters

The Na^+^ dependent Cl^−^/HCO_3_^−^ exchanger, NCBE (s*lc4a10*), is expressed in the basolateral membrane of the CPE (Figures 1, 2) (Praetorius et al., 2004a), where it performs Na^+^-dependent Cl^−^/HCO_3_^−^ exchange (Wang et al., 2000; Damkier et al., 2010a), although the precise mode of action differs between expression systems (Parker et al., 2008). In all cases NCBE mediates the transport of Na^+^ and HCO_3_^−^ into the cell. In rat and human the electroneutral Na^+^:HCO_3_^−^ co-transporter, NBCn1 is also found in the basolateral membrane of the CPE (Praetorius and Nielsen, 2006). The Na^+^ dependent Cl^−^/HCO_3_^−^ exchanger (*slc4a8*) is expressed in the basolateral membrane of the rat fetus (Chen et al., 2008a), however, in adult rats the expression is absent suggesting a role of NDCBE in choroid plexus development.

### SLC4a10

The Na^+^ HCO_3_^−^-importer, NCBE, was originally cloned and functionally characterized in 2000 (Wang et al., 2000). NCBE is primarily located in the CNS (Giffard et al., 2003).

A knockout mouse model of the *slc4a10* gene was generated by Jacobs *et al* (Jacobs et al., 2008) by introducing a targeted deletion of exon 12, encoding the first trans-membrane region of NCBE, causing a frame-shift and a premature stop codon in exon 13.

Disrupting NCBE has profound structural implications in the CPE; microvilli are reduced, the intercellular space of the CPE is enlarged (Jacobs et al., 2008), and interestingly, the polarization of NHE1 changes from the apical to the basolateral membrane of the cells (Damkier et al., 2009). These changes may be caused by the redistribution of ezrin (Damkier and Praetorius, 2012), an anchoring protein important for e.g., cell shape and the organization of membrane proteins (Jiang et al., 2013), including the binding of NHE1 to the actin cytoskeleton (Denker et al., 2000). The complete mechanism and consequences of these changes, however, remains to be fully elucidated. NCBE is central for the secretory function of the CPE. NCBE knockout mice displayed 80% smaller brain ventricle volumes (Jacobs et al., 2008), most likely due to a decrease in CSF production, and thereby supporting the hypothesis of NCBE as the bottleneck for basolateral Na^+^-loading in the CPE. Loss of NCBE was also found to decrease expression levels of Na/K-ATPase and AQP1 in the CP (Damkier and Praetorius, 2012), further supporting a greatly reduced CSF production capacity. In comparison, disruption of AQP1 alone only lowers CSF production by 25% (Oshio et al., 2003). In isolated CPE cells from the knockout mice there is an 80% reduction in the Na^+^ and HCO_3_^−^-dependent pH_*i*_ recovery rate indicating a role of NCBE in CPE pH_*i*_ regulation and perhaps movement of HCO_3_^−^ from blood to CSF. As in the NBCe2 knockout studies the compensatory changes in the proteins important for CSF secretion and pH regulation the interpretation of the role of NCBE in the CSF secretion and warrants the need for further studies to highlight this task.

Expression of NCBE is also found in the retina (Hilgen et al., 2012). Visual impairment, in terms of decreased contrast sensitivity, retinal displacement, and atypical electro-retinograms were reported in mice with NCBE disruption.

As mentioned earlier, neuronal firing activity alters both intra- and extracellular pH, which in turn modifies neuronal excitability (Chesler and Kaila, 1992). NCBE is also widely expressed in the neurons of the hippocampus, cortex and cerebellum (Chen et al., 2008b), and is presumably involved in the pH homeostasis of the brain (Chen et al., 2007). Its role in regulating pH with regard to modifying neuronal excitability, is therefore of particular interest. Indeed, NCBE knockout mice held a higher threshold to pentylenetrazol, with longer latency of seizure onsets as well as decreased seizure severity (Jacobs et al., 2008).

Human studies involving NCBE are also mainly associated with changes in neuronal excitability. In a patient with partial, complex epilepsy and cognitive dysfunction, genetic analysis revealed a translocation with a break point between exon 2 and 3 of *SLC4A10* (Gurnett et al., 2008). The epilepsy in this patient is in contrast to what was seen in the knockout mice that seem to be protected against seizures. Another case study of a patient with generalized epilepsy and speech retardation, found a deletion in the 2q24.2 region, which includes the *SLC4A10* locus (Krepischi et al., 2010). Finally, statistical analysis of a large cohort of patients with autism, revealed a single deletion of exon 1 of the *SLC4A10* in a pair of monozygous autistic twins (Sebat et al., 2007). The twins were, however, not further described, and whether they also suffered from epilepsy is therefore unknown.

## Luminal Na^+^ dependent acid-base transporters

The *slc4a5* gene product, NBCe2, is expressed in the luminal membrane of the CPE (Figure 2; Bouzinova et al., 2005). NBCe2 mediates the transport of three HCO_3_^−^ together with one Na^+^ from the cell to the CSF. This stoichiometry suggests a role of NBCe2 in CSF pH regulation (Millar and Brown, 2008). In mouse, and in some cases in humans, NBCn1 (*slc4a7*) is expressed in the luminal membrane (Praetorius and Nielsen, 2006). The apparent discrepancy in localization of this protein between species could indicate that the protein in CPE is most likely involved in protecting the cell from intracellular acidification rather than in secretion of CSF as the direction of transport regardless of the membrane expression is most likely inwards. The Na^+^ Borate transporter (*slc4a11*) is similarly expressed in the luminal membrane of CPE (Damkier et al., 2007). This transporter was recently shown to transport Na^+^ in exchange for H^+^ suggesting a similar role as the Na^+^/H^+^ exchanger, NHE1, in the luminal membrane. Finally, NHE1 (*slc9a1*) is present in the luminal membrane of the CPE (Damkier et al., 2009). The localization of NHE1 indicates that this protein, similar to NBCe2, could be an important mechanism for regulating CSF pH.

In addition to these transporters the CPE expresses numerous other transporters involved in the secretion of solutes and nutrients. These are outside the scope of this review but are reviewed elsewhere (Damkier et al., 2010b; Ho et al., 2012).

### SLC4a5

The *slc4a5* gene product, NBCe2 (also known as NBC4), is an electrogenic sodium bicarbonate co-transporter. It exists in at least two variants, NBCe2a (1137-residue polypeptide), and NBCe2c (1074-residue polypeptide). In choroid plexus a novel variant, NBCe2g, was reported (Fukuda et al., 2013). The variants differ in their C-termini (Pushkin et al., 2000). When expressed in mammalian cells, NBCe2 mediates DIDS-sensitive sodium-bicarbonate co-transport (Virkki et al., 2002; Romero et al., 2004). In CPE, NBCe2 mediates the efflux of one Na^+^ together with three HCO_3_^−^ from the cell into the CSF (Millar and Brown, 2008). When expressed in Xenopus laevis oocytes, NBCe2 shows a 2:1 HCO_3_^−^:Na^+^ stoichiometry (Virkki et al., 2002). Thus, the stoichiometry of NBCe2 appears to be cell specific. In addition to the choroid plexus, NBCe2 has been localized in kidney, liver, and gastrointestinal tract although the precise subcellular localization of the protein is not known (Sassani et al., 2002; Xu et al., 2003).

Currently, there are three published *slc4a5* mouse models. The first *slc4a5* knockout mouse was generated by infecting embryonic stem cells with a retroviral gene trap vector, that integrated upstream of exon 15 (Kao et al., 2011) inserting a stop codon that prevented further translation. In the predicted protein sequence this should truncate the protein after 650 amino acids after the predicted fifth trans-membrane domain. The second mouse model was generated by deleting the seventh coding exon of the *slc4a5* gene. This deletion lead to the loss of the N-terminal part of the protein and a truncation due to a frame-shift in the open reading frame of the transcript before the putative first trans-membrane domain (Groger et al., 2012). The third model was generated by deleting exon 13, which removed codons 474–563 and created a frame-shift mutation. By creating the frame-shift, the potential for translation of the 500 amino acid membrane-spanning domains of the protein was eliminated (Chen et al., 2012). Multiple defects in the central nervous system were observed in the *slc4a5* deficient mice. The defects include decreased volume of lateral brain ventricles, decreased intracranial pressure, changes in CPE cell morphology, and alterations of the subcellular distribution of *slc4a10* and Na, K ATPase subunits (Kao et al., 2011). The mice displayed changes in CSF composition (decreased [HCO_3_^−^] and increased [K^+^]) as well as decreased seizure susceptibility upon administration of the pro-convulsant drug pentylenetetrazol. The data from these mice indicate that NBCe2 is important for CSF secretion and pH regulation; however, the compensatory changes in the localization of transporters equally imperative for CSF secretion and pH regulation make it difficult to determine the specific role of NBCe2. In addition to the CPE phenotype, vision was also affected due to retinal detachment and optical nerve changes in these mice (Kao et al., 2011).

The second *slc4a5* knockout mouse was found to be hypertensive and presents with metabolic acidosis (Groger et al., 2012). This phenotype was attributed to the renal expression of NBCe2. Investigations of the levels of proteins expressed in the kidney revealed an increase in other bicarbonate transporters including AE1, Pendrin, and *slc4a7*. Proteins involved in Na+ handling in the kidney were not investigated. The brain ventricle volume was not reduced in this knockout mouse (Groger et al., 2012) highlighting a discrepancy between the two mouse models.

In the third *slc4a5* knockout mouse the duodenum was investigated. There was no apparent difference in HCO_3_^−^ secretion in the knockout mouse compared to wild-type suggesting that NBCe2 does not play an important role in duodenum (Chen et al., 2012). Several studies have linked polymorphisms in *SLC4A5* to salt-sensitive hypertension in humans (Barkley et al., 2004; Carey et al., 2012). It is not known whether these polymorphisms also associate to CSF disorders or visual defects.

### SLC4a7

The electroneutral sodium bicarbonate co-transporter, NBCn1 (also known as NBC3), was originally cloned in three variants from rat smooth muscle cells (Choi et al., 2000). Since then, more variants have been cloned and now 32 *slc4a7* products encoding full-length transporters exist (Liu et al., 2013; Parker and Boron, 2013). NBCn1 functions as an electroneutral Na^+^:HCO_3_^−^ co-transporter with a stoichiometry of 1:1 and is, compared to other Na^+^:HCO_3_^−^ transporters, less sensitive to DIDS (Choi et al., 2000).

NBCn1 has been localized in many tissues including CPE (Praetorius et al., 2004a), renal thick ascending limb (TAL) (Odgaard et al., 2004) and medullary collecting duct epithelial cells(Praetorius et al., 2004b), vascular smooth muscle cells and endothelial cells from a broad range of blood vessels(Boedtkjer et al., 2008). The function of NBCn1 has been investigated in various cell types. In kidney, NBCn1 expression increases in the basolateral membrane of TAL following chronic metabolic acidosis (Odgaard et al., 2004). Apart from being a cellular pH regulator NBCn1 may be involved in maintaining medullary transcellular NH^+^_4_ -shuttling by maintaining a favorable TAL pH_i_. In duodenal villus enterocytes NBCn1 has a prominent role as a major pH_i_ regulatory mechanism (Praetorius et al., 2001; Chen et al., 2012).

NBCn1 is expressed by the CPE in rodents and humans (Praetorius et al., 2004a; Damkier et al., 2006, 2007), but the subcellular distribution is not fully clarified. In rat and human (Praetorius et al., 2004a; Praetorius and Nielsen, 2006), NBCn1 antibodies localize the transporter to the basolateral domain of the CPE. In human and some mice strains, however, NBCn1 is found in the luminal membrane domain (Damkier and Praetorius, 2012). Considering the ions transported by NBCn1, it is plausible that NBCn1 is important in pH-regulation of the CPE. The HCO_3_^−^ dependent pH_*i*_-regulation at steady-state level and after cellular acidification has been investigated in order to describe the function of the Na^+^:HCO_3_^−^ transporters in pH-regulation in the CPE cell (Bouzinova et al., 2005). The study shows a partially DIDS-sensitive Na^+^ dependent HCO_3_^−^ uptake in rat CPE cells. The DIDS-insensitive component may be mediated by NBCn1.

Currently, there are two known *slc4a7* mouse models. The first knockout mouse was generated using a targeting vector causing a deletion of parts of exon 5 which lead to a truncation of the protein after 137 amino acids (Bok et al., 2003). The second mouse model was generated using a gene trap vector integrated 434 bases upstream of the MEAD start codon of *slc4a7* (Boedtkjer et al., 2011).

The first *slc4a7* knockout mice showed characteristics of Usher syndrome type II which includes moderate to severe, progressive hearing loss and normal vestibular function. The major consequence of NBCn1 deletion within the inner ear is the selective loss of inner and outer hair cells as well as the loss of supporting cells from the hook region. A disruption in the electroneutral sodium bicarbonate flux mediated by NBCn1 cause a disruption of the pH sensitive K^+^ secretion into the endolymph. In the second mouse, vascular smooth muscle cells of the NBCn1 knockout mouse had a lower steady state pH_i_, resulting in a disruption in artery function and blood pressure regulation (Boedtkjer et al., 2011).

Furthermore, genome wide association studies have indicated that single nucleotide polymorphisms (SNPs) in the 3′ UTR of NBCn1 are linked to susceptibility to breast cancer (Ahmed et al., 2009). It is still speculative in what way NBCn1 contributes to the etiology of breast cancer (Parker and Boron, 2013). Additionally SNPs NBCn1 was linked to blood pressure dysregulation (International Consortium for Blood Pressure Genome-Wide Association et al., 2011).

### SLC4a11

*Slc4a11* was cloned in 2001 (Parker et al., 2001) and originally characterized as a 2 Na^+^/B(OH)^−^_4_ co-transporter and, in the absence of borate, as an electrogenic Na^+^/2OH^−^ co-transporter (Park et al., 2004). A more recent characterization suggests that *slc4a11* has EIPA-sensitive Na^+^:OH^−^(H^+^) permeability and does not transport either HCO_3_^−^ or borate (Ogando et al., 2013)

Two *slc4a11* knockout mice have been described. The first was generated by a retroviral gene trap vector integrated upstream of exon 2 of *slc4a11* containing a stop codon (Lopez et al., 2009). The second was generated using a targeting vector causing fusion of exon 10 to beta-galactosidase and deletion of exons 11–18 which leads to truncation of the gene before the first predicted trans-membrane domain (Groger et al., 2010).

Both mice have been thoroughly investigated with respect to their corneal phenotype. The two mice seemingly differ in respect to the severity of the phenotype. The first mouse displayed no corneal changes but showed a collapsed membranous labyrinth that encases the sensory epithelia as well as disruption of the neural transduction at receptor level. The second mouse showed thickening of the endothelial cell layer, Descemets membrane, stroma, and the endothelial cell layer of the cornea as well as increased [Na^+^] in corneal stroma. The mice potentially differ because the first study was performed in young mice and the latter in 12 months old mice. In the second study, the renal phenotype was also studied and the mice presented with polyuria, loss of NaCl in the urine and hypoosmolar urine.

*Slc4a11* is localized in the luminal membrane of human CPE (Damkier et al., 2007). The physiological role of *slc4a11* in the CPE is not known. The recent functional study of *slc4a11* as an EIPA-sensitive Na^+^/H^+^ exchanger indicates a role as an acid extruder. In NHE1 knockout mice, however, there is no evidence of Na^+^ dependent pH_i_ recovery in the absence of CO_2_/HCO_3_^−^ in isolated CPE (Damkier and Praetorius, 2012) suggesting that NHE1 is the only active Na^+^/H^+^ exchanger and that *slc4a11* does not play any role for pH_i_ regulation at least in the normal physiological range. However, in Ncbe knockout mice where NHE1 is basolateral a luminal HCO_3_^−^ independent Na^+^, base-importer becomes active in the absence of NHE2-4 expression. It cannot be ruled out that *slc4a11* protein is involved in this transport phenomenon.

In humans, mutations in *SLC4A11* are associated with congenital hereditary endothelial dystrophy (CHED), corneal dystrophy and perceptive deafness (Harboyan syndrome) and late onset Fuchs endothelial corneal dystrophy (FECD) (Desir et al., 2007).

### SLC9a1

NHE1 is an amiloride-sensitive Na^+^/H^+^-exchanger transporting one Na^+^ into the cell in exchange for one H^+^ and thus the protein has a function similar to the inwardly directed Na^+^ dependent HCO_3_^−^ co-transporters, which is net acid extrusion. NHE1 is ubiquitously expressed in all mammalian cells (Sardet et al., 1988). In epithelial cells, NHE1 is usually expressed in the basolateral membrane, as seen in certain renal tubular cells (Biemesderfer et al., 1992).

NHE1 is involved in acid extrusion in e.g., neurons (Yao et al., 1999), renal TALs (Good et al., 2004), parotid glands (Evans et al., 1999), choroid plexus (Damkier et al., 2009), and vasculature (Boedtkjer et al., 2012a). The activity of NHE1 is also a critical factor in regulation of cell motility and cell volume (Boedtkjer et al., 2012b).

Currently, there are two known *slc9a1* mouse models. One model was made by targeting the sixth and seventh trans-membrane-spanning domains of *slc9a1* (Bell et al., 1999) while the other, the slow-wave epilepsy (*swe*) mutant arose as a spontaneous mutation (Cox et al., 1997). There are phenotypic similarities between the targeted Nhe1^−/−^ mutant and the *swe* mutant. The mice show ataxic gait at 2 weeks of age and increased neuronal excitability in neonates. The mutants showed increased mortality even before weaning and a postmortem appearance suggestive of death by convulsive seizures. NHE1 mRNA in CPE has been shown in human, rat and pig (Kalaria et al., 1998). It was first suggested that NHE1 was expressed in the basolateral membrane of CPE cells as it is in other epithelial cells. This was supported by studies showing a 50% decrease in CSF secretion when the NHE1 inhibitor, amiloride, was given intravenously (Davson and Segal, 1970). NHE1 was later immunolocalized to the luminal membrane domain in mouse and human CPE and functionally found to be the only active HCO_3_^−^ -independent, Na^+^-dependent acid extruder in mouse CPE (Damkier et al., 2009).

The significance of NHE1 expression in CPE is not fully understood. The subcellular location in the luminal membrane could indicate that it may function as a pH_*i*_ regulator, although the expression level seems to be quite low. In NCBE knockout mice, NHE1 translocates to the basolateral membrane and may in this situation function as a Na^+^ loader to sustain the lowered CSF secretion (Damkier et al., 2009).

No studies have shown a correlation between NHE1 disruption and human disease, but it is clear that NHE1 is an important pharmacological target in some pathophysiological situations as ischemic heart disease and human breast cancer (Malo and Fliegel, 2006).

## Choroid plexus as target for pharmacological treatment

**Figure 3:**
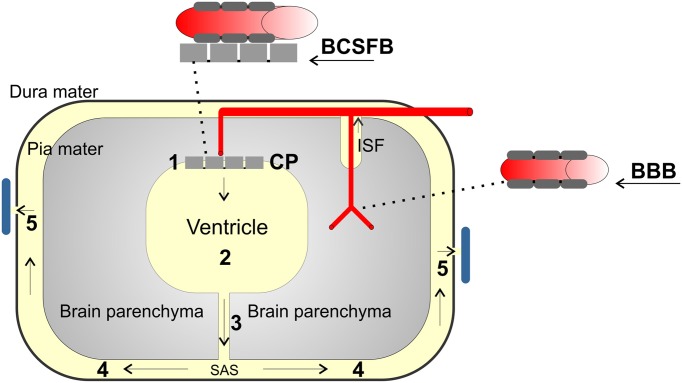
**Schematic presentation of the circulation of CSF and the barriers of the brain.** CSF is mainly produced by the choroid plexus (1, CP) in all four ventricles of the brain 2. The CSF is drained via narrow foramina 3 into the subarachnoid space (SAS, 4) between the pia mater and the dura mater. The CSF is continuously returned to the circulation mainly via the arachnoid granulations 5. Some CSF is also generated by the interstitial fluid (ISF) arising from the blood vessels of the BBB and the ependyma. The blood brain barrier (BBB) is made from the endothelial cells in the brain capillaries. Permeability for solutes to the brain is limited by this barrier that is kept very constricted by tight junctions. The blood-CSF barrier (BCSFB) consists of relatively leaky capillaries but the tight junctions of the epithelial cells restrict the permeability to the CSF.

Delivery of drugs from blood to the brain is greatly limited by the tightness of the barriers in the brain, the BBB and the BCSFB. The CPE is the key component in the BCSFB. The physical tightness of the BCSFB is accomplished by the tight junctions connecting the epithelial cells, whereas the tightness of the larger BBB is made by the tight junctions between the endothelial cells in the brain capillaries (Figure 3; Reese and Karnovsky, 1967). In addition to the physical tightness, both the BCSFB and especially the BBB contain ATP binding cassette (ABC) transporters that actively rid the brain of xenobiotics thereby further preventing drug-delivery to the brain. Therapeutic strategies have targeted these transporters to increase delivery to the brain through the BBB (Hartz and Bauer, 2010). In some cases, injections of therapeutics directly into the subarachnoid space (intrathecal administration) are used as targeted treatments of diseases in the brain such as tumors and infections (Varelas et al., 2008; Serwer and James, 2012).

As the physical barrier function of the BCSFB is not in the vasculature but rather in the CPE cells the proteins in the basolateral membrane of CPE could potentially be targeted in therapy to inhibit CPE functions, e.g., secretion and pH regulation of CSF. Given the dramatic effects on CSF secretion of knocking out NCBE, this protein is an obvious drug target for reducing CSF secretion. Direct neuronal side effects of inhibition of NCBE may not be relevant since the protein within the CNS would be protected by the BBB and thereby only the CPE would be targeted. However, brain pH could be affected through equilibration with a possibly altered CSF pH. The brain is covered by the rigid skull which necessitates a tight regulation of volume and thereby intracranial pressure (ICP) in order to avoid damage to the brain tissue. Sudden increases in ICP can in the worst case lead to fatal incarceration. Overall, increases in volume inside the skull can be placed within the three “compartments” of the brain; the brain tissue (tumors), the blood (hemorrhage, swelling following ischemia or trauma) or the CSF (hydrocephalus). According to the Monro-Kelli doctrine, any increase in one of these compartments needs to be balanced by a decrease in another (Mokri, 2001). Decreasing CSF secretion during the acute stages of increases in ICP could be extremely beneficial for the patient outcome.

Regulation of CSF pH and thereby brain pH is essential for normal brain function and is similarly important for inhibition of seizures. Both the NCBE and NBCe2 knockout mice seem to have a higher seizure threshold than wild type. It is known that lowering of pH during a seizure causes an activation of the acid sensing Na^+^ channel, ASIC1a, leading to an inhibition of neuronal firing and thereby cessation of the seizure (Ziemann et al., 2008). The loss of a bicarbonate transporter in the NBCe2 and NCBE knockout mice most likely causes a decrease in CSF-pH which could potentially be protective for the brain. In addition to inhalation of CO_2_ in the acute stages of a seizure these proteins could be potential targets in the long-term treatment of seizure disorders by acutely lowering CSF pH and thereby brain pH.

## Conclusions

The study of choroid plexus physiology by use of knockout mice targeting the Na^+^ dependent acid-base transporters of the *slc4* and *slc9* families have so far highlighted many aspects of the significance of these transporters in CSF secretion and pH regulation. Further studies are warranted for highlighting the role of *slc4a11* and *slc9a1* in choroid plexus biology.

The studies highlight the importance of epithelial transporters *in vivo*, however the findings in genetically modified mice include the compensatory mechanisms that take place during fetal development and early life, and call for a general need for caution when interpreting these data. The genetic removal of some transporters, such as NCBE and NBCe2 leads to restructuring of the entire epithelium. This is interesting from a cell structure point-of-view but when investigating the particular protein of interest these compensatory changes could also completely change cell function and are thus not specific to removal of one protein. The compensatory changes could potentially be minimized by either creating an inducible knockout mouse allowing the mouse to fully develop before inducing the deletion, by generating mice that carry a specific mutation that blocks the NBC action, or by intraventricular installation of siRNA targeting the gene product. These kinds of studies introduce other complicating factors including the success rate of knockdown. This emphasizes the need for selective inhibitors both for studying the transporters of the *slc4* family but also for use in the pharmacological targeting of the transporters in disease.

## Author contributions

The manuscript was written with equal contributions from all authors. Final editing was mainly carried out by Henriette L. Christensen and Helle H. Damkier. Figures were created by Helle H. Damkier.

### Conflict of interest statement

The authors declare that the research was conducted in the absence of any commercial or financial relationships that could be construed as a potential conflict of interest.
